# COVID-Kavach-Based Seropositivity in the General Population of Ahmedabad: Just Before the Start of the Vaccination for the Elderly in India

**DOI:** 10.7759/cureus.22759

**Published:** 2022-03-01

**Authors:** Om Prakash, Bhavin Solanki, Jay K Sheth, Milan Nayak, Mina Kadam, Sheetal Vyas, Aparajita Shukla, Hemant Tiwari

**Affiliations:** 1 Indian Administrative Services, Ahmedabad Municipal Corporation, Government of Gujarat, Ahmedabad, IND; 2 Health Department, Ahmedabad Municipal Corporation, Ahmedabad, IND; 3 Community Medicine, Ahmedabad Municipal Corporation Medical Education Trust Medical College, Ahmedabad, IND; 4 Microbiology, Ahmedabad Municipal Corporation Medical Education Trust Medical College, Ahmedabad, IND; 5 Community Medicine, Nathiba Hargovandas Lakhmichand (NHL) Municipal Medical College, Ahmedabad, IND

**Keywords:** sars-cov-2, immunity, covid-kavach, sero-surveillance, igg antibody, covid-19

## Abstract

Background

The present study was carried out in succession of three serosurvey studies carried out during 2020 in Ahmedabad with an objective to estimate the seroprevalence of immunoglobulin G (IgG) antibodies against severe acute respiratory syndrome coronavirus 2 (SARS-CoV2) in Ahmedabad city so as to scientifically understand the pandemic progression.

Methods

Polio booth-based stratification was followed for the population-based stratified sampling among the general population of Ahmedabad. The seroprevalence was compared with various factors for valid and precise predictions regarding the immunity status of the population.

Results

As on February 2021, the seroprevalence for IgG antibodies against SARS-CoV2 in the general population of Ahmedabad was 27.92% (95% confidence interval 27.06-28.80), much below the minimum desired for herd immunity. Comparison of seropositivity with age groups showed higher seroprevalence with increasing age groups. Seroprevalence was higher among males (29.08%) than females (27.01%) and the difference was statistically significant (Z=2.30, P=0.02). Calculating the seropositivity among the subcategories, cases had a seropositivity of 64.90% and family contacts had a seropositivity of 28.00%. Seronegative cases indicate the possibility of absent, undetectable, or disappearing IgG antibodies. Seropositivity of 37% among the vaccinated individuals may be related to dose and duration of vaccination, as the COVID vaccination had started just before the present study and none had completed 14 days after the second dose.

Conclusions

The low level of IgG antibodies against SARS-CoV2 using the COVID-Kavach test kit in the general population of Ahmedabad city of India, as on February 2021, before the start of COVID vaccination for the general population suggests that the preventive measures be strongly followed for continued control of the pandemic situation at least till majority of the population is effectively covered with vaccination.

## Introduction

With reportedly nearly 7 million population, Ahmedabad city of Gujarat is a highly populated megacity in the western part of India [1]. Right from the start of pandemic, Ahmedabad was one of the highly affected cities, as during April 2020, the prevalence of COVID-19 in the containment zone of Ahmedabad was highest at 55%, as reported in a study conducted by Indian Council of Medical Research (ICMR) [2]. For a newly identified infectious agent, like severe acute respiratory syndrome coronavirus 2 (SARS-CoV2), World Health Organization (WHO) has also suggested monitoring through seroprevalence [3]. Serosurvey, as defined by WHO, is collection and testing of serum from a sample of a defined population over a specified period of time to estimate the prevalence of antibodies against a given specific infectious pathogen as an indicator of immunity [4]. Seroprevalence, on the other hand, is the proportion of people in a population who test seropositive for a specific infectious pathogen. Surveillance through serological survey helps in understanding the pandemic in a better way [5]. The ICMR, in its document dated 30th May 2020, has issued directives to conduct serosurvey along with influenza-like Illness and severe acute respiratory infection surveillance [6]. Acting upon these guidelines, the health department of the Ahmedabad Municipal Corporation (AMC) has already completed three serosurveys during 2020 in the months of June, August, and October. Apart from the general population, the second and third serosurveys also covered cases, contacts, and healthcare workers (HCWs) as additional separate categories and all these results have been documented widely [7-16]. The seroprevalence for the immunoglobulin G (IgG) antibodies against SARS-CoV2 among the general population in these three serosurveys was 17.61%, 23.24%, and 24.20%, respectively [7,8,13]. The previous serosurveys highlighted the progression of the pandemic in the general population and the risk dynamics among cases, contacts, and HCWs.

Ahmedabad had reported major peak of COVID-19 cases after the last serosurvey in October 2020. So, to assess the seropositivity in the general population, the health department of the AMC planned and carried out the fourth serosurvey during the last week of February 2021 using the same testing kit, i.e., COVID Kavach. Although COVID vaccination had just started from January 16th, 2021 for the HCWs and later on for the frontline workers (FLWs), the vaccination for the general population had not started by the time of the present study. So, the timing of the present study was ideally suited with regard to vaccination for the general population.

The objective of the study was to estimate the seroprevalence in the general population of Ahmedabad, just before the vaccination for the general population, so that it can serve as baseline to study any change in seropositivity during or after vaccination.

## Materials and methods

Study setting, population, and duration

The present study was carried out in Ahmedabad city of Gujarat, India. The study population is the general population of Ahmedabad city. The sample collection started from 22nd February 2021 and completed by 1st March 2021 before the vaccination for the elderly general population started.

Ethical considerations

The study was carried out after the approval by the Institutional Ethics Committee of the Ahmedabad Municipal Corporation Medical Education Trust (AMC MET) Medical College. Participants from general population were enrolled after informed written consent and full confidentiality was ensured at all possible levels.

Sampling method

Ahmedabad city has recently grown bigger, with new peri-urban area added within the city limits. The Pulse Polio National Immunization Day round was also conducted in the recent past on 31st January 2021. The pulse polio micro-plan was recently updated, represents all strata of the community, and booth locations are distributed according to the population proportion. So, the sample population representative to the general population was selected using the stratified sampling and the pulse polio booths were used for the purpose of stratification.

Sample size calculation

The results from the earlier serosurveys showed that some of the urban primary health centers (UPHCs) had seroprevalence of nearly 50% [6,7,12]. So, for sample size calculation, we considered 50% prevalence in a population of 70 lakhs, a confidence level of 95%, and the margin of error as 1%. Our estimated minimum sample size was calculated to be around 9600.

Sampling procedure

Ahmedabad city has around 3400 table polio booths (excluding the mobile and transit booths) across seven zones of Ahmedabad city with defined teams and localities that the booth covers. Polio booths are enlisted UPHC wise, and so from each UPHC polio booth list, alternative polio booths were selected for the purpose of sampling. To get a little higher sample size than the target, odd number booths were selected rather than even number booths (so that one additional polio booth area is selected if the total number of booths for the UPHC is having an odd number). This translates into a minimum of six samples from each of the selected polio booth. Convenience sampling was used for the selection of houses from the pulse polio booth field area. From each house one person giving informed written consent for enrollment was selected. Thus, six individuals from six different houses were enrolled from each of the selected pulse polio booth field area and their samples were collected.

Inclusion and exclusion criteria

Any individual from the randomly selected house from the selected polio booth field area was eligible to be included in the study, without any restrictions for age or sex. Children less than 18 years were also enrolled with the informed written consent and assent of their parents/caretaker. The exclusion criteria did not include HCWs or FLWs and so even vaccinated individuals (a few HCWs or FLWs) were also enrolled as part of the study. Thus, the required sample representative to the general population was selected for the purpose of serosurvey.

Data collection

In the field area, laboratory technicians posted at the UPHC, along with the local HCWs, collected the blood samples from the enrolled participants after an informed written consent and a brief data collection tool was filled for the purpose of data collection. Apart from the personal and demographic information, the study questionnaire included questions regarding vaccination, COVID-19-positive test in the past, and COVID-19-positive test in any of the family members.

Testing kit and testing methodology

Zydus Diagnostics has developed and manufactured the “COVID Kavach” (anti-SARS CoV-2 IgG antibody detection enzyme-linked immunosorbent assay (ELISA)) kits. This kit is already approved by the ICMR after validation from the National Institute of Virology, Pune, India. The earlier serosurveys in Ahmedabad were carried out using the COVID-Kavach test kit. With the reported sensitivity of 92.37% and specificity of 97.9% the documented results of COVID-Kavach test kit are highly reliable [17]. The manufacturer reported no cross-reactivity with RT-PCR-confirmed positive serum for other viral infections. Accredited laboratories carried out the sample testing. These laboratories have all necessary equipment and resources, maintain the standards of quality testing, and routinely undertake external quality assurance assessment. Testing procedures were followed as per the manufacturer’s instructions. For each plate, samples with optical density (OD) value more than the cutoff value and positive/negative (P/N) ratio more than 1.5 were considered as positive. Samples with OD value of 10% ± ranges of the cutoff were considered to be indeterminate. The P/N ratio was defined as the ratio of average OD value of the positive control divided by the average OD of the negative control. The cutoff OD value was calculated as the average OD value of negative control +0.2.

Data analysis

Epi-info software (https://www.cdc.gov/epiinfo/index.html) and Microsoft Excel were used for the purpose of data analysis including proportions and Z-test.

## Results

From 10,157 total collected samples, 21 samples were rejected and results were available for 10,136 samples (Table 1). The result shows that the crude seropositivity for the general population of Ahmedabad, as on February 2021, is 27.92% (95% confidence interval (CI) 27.06-28.80). Results were available for 4471 male and 5665 females. A total of 1300 male demonstrated the presence of IgG antibodies, giving the positivity of 29.08% (95% CI 27.76-30.42). A total of 1530 female demonstrated the presence of IgG antibodies, and their positivity was 27.01% (95% CI 25.87-28.18). The males had higher seropositivity than females and their difference was statistically significant (Z=2.30, P=0.02).

**Table 1:** Analysis of COVID-19 serosurvey positivity in the general population of Ahmedabad CZ: Central Zone; EZ: East Zone; NWZ: North West Zone; NZ: North Zone; SWZ: South West Zone; SZ: South Zone; WZ: West Zone

	Female			Male			Total			95% Confidence Interval
	Results	Positive	% Positivity	Results	Positive	% Positivity	Results	Positive	% Positivity	
Gender	5665	1530	27.01	4471	1300	29.08	10136	2830	27.92	27.06-28.80
Age group										
0-9	7	0	0.00	15	2	13.33	22	2	9.09	01.12-29.16
10-19	255	53	20.78	234	54	23.08	489	107	21.88	18.44-25.76
20-29	1837	378	20.58	1045	225	21.53	2882	603	20.92	19.48-22.45
30-39	1289	327	25.37	985	282	28.63	2274	609	26.78	25.00-28.64
40-49	972	319	32.82	788	251	31.85	1760	570	32.39	30.24-34.61
50-59	705	248	35.18	611	219	35.84	1316	467	35.49	32.95-38.11
60-69	431	155	35.96	548	188	34.31	979	343	35.04	32.11-38.08
70-79	151	41	27.15	211	65	30.81	362	106	29.28	24.83-34.17
80-89	17	8	47.06	33	14	42.42	50	22	44.00	29.99-58.75
90-99	1	1	100.00	1	0	0.00	2	1	50.00	01.26-98.74
Zone										
CZ	598	139	23.24	529	163	30.81	1127	302	26.80	24.29-29.46
NZ	1054	265	25.14	779	181	23.23	1833	446	24.33	22.42-26.35
EZ	1083	324	29.92	687	228	33.19	1770	552	31.19	29.07-33.38
SZ	1164	349	29.98	610	196	32.13	1774	545	30.72	28.62-32.91
SWZ	436	101	23.17	414	107	25.85	850	208	24.47	21.70-27.47
WZ	812	218	26.85	861	251	29.15	1673	469	28.03	25.93-30.29
NWZ	518	134	25.87	591	174	29.44	1109	308	27.77	25.22-30.48

**Figure 1:**
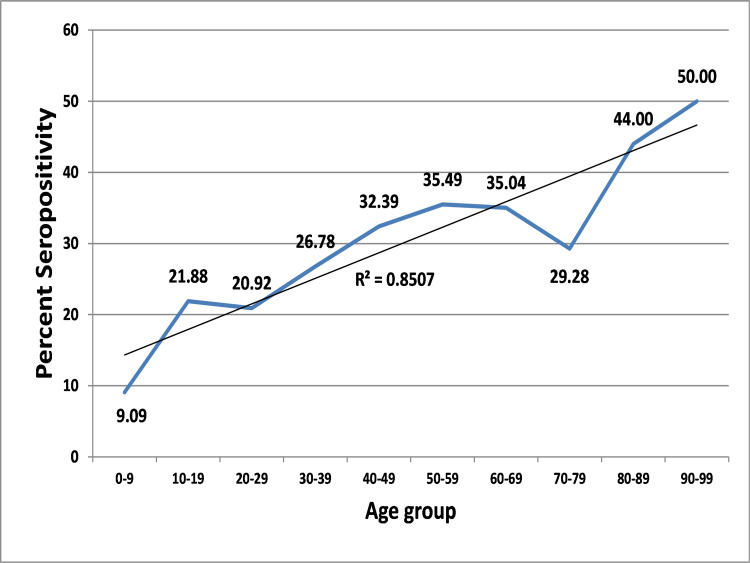
Age-group-wise seropositivity in the general population in Ahmedabad

The age-wise analysis shows that the age of the enrolled individuals ranges from 2 to 90 years with a mean of 39.06±15.44 years, mode of 30 years, and median of 36 years. Seropositivity in the general population according to the age group (Figure 1) shows that the range of seropositivity was 9.09%-50.00%. The seropositivity is comparatively low among younger population. Comparison of seropositivity with age groups showed higher seroprevalence in age groups with higher age.

Comparison of seroprevalence according to zone shows that the variations are in the range of 24.33%-31.19%. The wider variation in zone-wise seropositivity recorded during the earlier serosurveys seem to be narrowing.

Seropositivity among cases, family contacts, and according to the COVID vaccination status is described below (Table 2).

**Table 2:** Category-wise seropositivity among the general population of Ahmedabad *Based on answers to question on vaccination. ^†^None completing 14 days after two doses (based on the starting point date of vaccination and study period). ^@^Based on the answer to question on whether a case of COVID-19. ^#^Based on not being a case and having a positive member in the family.

	Female			Male			Total		
Category	Results	Positive	% Positivity	Results	Positive	% Positivity	Results	Positive	% Positivity
Vaccinated^†^	195	74	37.95	132	47	35.61	327	121	37.00
Non-vaccinated*	5470	1456	26.62	4339	1253	28.88	9809	2709	27.62
Cases^@^	157	90	57.32	202	143	70.79	359	233	64.90
Family contact^#^	103	33	32.04	97	23	23.71	200	56	28.00

Seropositivity in vaccinated individuals

Based on the answer to the question regarding vaccine, the seropositivity among vaccinated individuals is 37.00% (95% CI 31.95-42.36).

Seropositivity in cases

Those individuals who replied “Yes” to the question on COVID-19-positive test since the pandemic were considered as cases without any cross-verification. Based on this the seropositivity among cases was 64.90% (95%CI 59.83-69.66).

Seropositivity among family contacts

When the enrolled individual is not a case, as per the earlier question, and there is a positive family member in their family, it means that the enrolled participant is a family contact of a case. The seropositivity among individuals having a family contact is 28.00% (95% CI 21.90-34.77).

## Discussion

Sero-surveillance studies are extremely useful in scientifically understanding the progress of the pandemic as well as the immunity status of the population. To monitor the growing burden of the pandemic, it is recommended to have repeated serosurveys [18]. There are various factors that markedly affect the seroprevalence [19]. That is why, understanding of various factors affecting immunity should be kept in mind while interpreting the results derived from the serological surveys.

Those who demonstrate presence of antibodies in their serum are seropositive individuals. Seroprevalence is an important indicator, which gives an approximate proportion of population with exposure to the infectious agent and gives an estimate of the cumulative incidence among population. This further helps in deciding the public health preventive measures for better control of the pandemic. Our study focuses on the seropositivity in the population of Ahmedabad as on end of February 2021, just before the start of vaccination for the elderly from the general population category.

The overall seropositivity among the general population at the end of February 2021 is 27.92%. This level of seropositivity also means that still a majority of the people of Ahmedabad are seronegative. Based on the derived results, it can be concluded that the preventive measures must be strongly followed for continued control of the pandemic situation. Ahmedabad reported a high number of cases during the last two months of the 2020 (and after the third serosurvey in October 2020). As compared to the previous serosurvey carried out during October 2020, there is an increase of 3.72% (from 24.20% to 27.92%) in seropositivity among the general population [13]. In view of the high number of cases recorded in the recent past (November-December 2020), the slight increase in the positivity requires further scientific research. This may be indicative of the temporary nature of the IgG antibodies which disappear or become undetectable after some time.

There is a higher number of females enrolled in the study. This is probably due to the fact that the enrollment and sample collection were carried out from the polio booth field area and working male population is less likely to be available. The difference in the seropositivity for both the sex groups is statistically significant. This is a finding that is different from other studies, which reported that the sex-wise difference is statistically not significant [20,21]. IgG-based seropositivity and the various factors affecting its seropositivity during the post-COVID period need to be studied in detail to find out the reasons for this statistically significant difference in the sex-wise seropositivity.

Analyzing the seropositivity according to the age groups shows that the seropositivity has an increasing trend with the increase in the age group. This may be because of the difference in the clinical presentation of the people from different age groups when they are COVID-19 positive. Published literature suggests that the young people are more likely to be asymptomatic or have milder clinical symptoms and their symptomatic phase is also more likely to stay for shorter duration [22-28]. Thus, scientifically it is documented that the presence, severity, and duration of the clinical symptoms are associated with better immune response. Since our study tool did not collect such data on the presence of symptoms as well as its severity, we are unable to confirm this finding in our study group of COVID-19 cases. However, the higher level of seropositivity in the participants with higher age group can possibly be explained with this association. Additional research studies may be carried out to generate greater as well as reliable evidences for understanding the scientific reasons for this trend.

The comparison of seropositivity among the seven zones of Ahmedabad shows that the zone-wise positivity has minor variations. As compared to the previous serosurvey studies, difference in the seropositivity across the zones has reduced. This suggests that the pandemic, which was initially focused on a limited area, gradually affected all the areas and has been generalized with community transmission.

COVID-19 vaccination started in India from 16th January 2021, just before the present study [29]. Initially, only HCWs were covered for the vaccination. Later on, from February 2021, FLWs were also included as beneficiary for the vaccination. The vaccination for the elderly population started from March 2021, just after the present survey. So, in the present sampling, we registered only a limited number of people (HCWs and FLWs from the general population) who had already received the vaccine during the initial phase of vaccination. Even among the vaccinated, none would have completed 14 days after their second dose as per the start of vaccination in India and our study duration. As our study tool did not record the number of doses, we could not compare the difference in seropositivity with doses, even if the number may be too small to compare. Among the vaccinated people (Table 2) we recorded the seropositivity of 37% among the vaccinated individuals. This low level of seropositivity may be because most of the individuals among the vaccinated would have received only one dose. Moreover, the time since vaccination is also important as immunity is reported to be 14 days after both the doses are received. None of the enrolled individuals would have completed 14 days after the second dose if at all they belong to HCW and had received two doses. The study findings with above facts may explain the lower level of seropositivity among the vaccinated individuals as recorded in our study.

As the study sampling was general population based, we registered a limited number of HCWs/FLWs in our study, restricting the numbers of vaccinated individuals in the present study. A total of 9809 (96.77%) from the total sample population were non-vaccinated. The seropositivity among the non-vaccinated group (27.62%) is very similar to the seropositivity in the general population (27.92%) as majority of enrolled individuals in the study have not yet received the vaccine.

For operational feasibility, a limited number of questions were included in our questionnaire. One of the questions was regarding the confirmed positive COVID-19 test in the past. The answer to this question helped in filtering cases from others. Among the 359 cases (Table 2), the IgG seropositivity was recorded in 233 individuals, giving a seropositivity of 64.90% (95% CI 59.83-69.66). Even though the cases have clear and documented exposure to the SARS-CoV-2, the seropositivity among cases is 64.90%. Such a finding indicates one of three possibilities. The first possibility is that all the cases might not be developing the IgG antibodies. The second possibility is that in many cases IgG may be present but their level might be undetectable. The third possibility is that the IgG antibodies might be disappearing at some point of time after the COVID infection. The real reason is still unclear but some scientific research studies are required to know the reasons for this low seropositivity among cases.

One of the questions to the enrolled individuals was related to a positive family member. When an individual is not a “case” and he/she reports a positive family member, it indicates that the enrolled individual is a family contact (high-risk contact) of a confirmed case of COVID-19. We recorded a total of 200 individuals with a history of family contact (Table 2). From these, a total of 56 (28%) demonstrated seropositivity. On account of their close contact, family members of the confirmed case of COVID are at higher risk of getting infection (and also the resultant antibodies). Seropositivity among family contacts of cases is 28%, which is slightly higher than that of the general population, i.e. 27.92%.

With very few individuals from case, family contact, or vaccinated category, majority of the study sample are non-vaccinated and neither a case nor a family contact. The seropositivity level among these individuals from the general population indicates that the pandemic has already affected one-fourth of the population in Ahmedabad. Many might have asymptomatic infection or have not been found positive on testing while symptomatic, but has resulted into demonstrable IgG antibodies.

Limitations

The reported age, vaccination status, case status as well as status as a family contact were not verified with any document and were recorded as replied by the enrolled participants. We did not collect the number of doses among the vaccinated individuals. Since the selection of the study participants was independent of their age and sex, the seropositivity among both the sex groups and various age groups should be interpreted with caution. Some of the characteristics of enrolled individuals may not have sufficient numbers to draw meaningful conclusions. Due to high sensitivity and specificity of the testing kit, we have ignored the slight variation in our actual estimate of seroprevalence.

## Conclusions

Seroprevalence based on the sero-surveillance in Ahmedabad city of India during February 2021, before the start of COVID vaccination for the general population, suggests that the level of IgG antibodies against SARS-CoV2 is low. It is recommended that the preventive measures be strongly followed for continued control of the pandemic situation at least till majority of the population is effectively covered with vaccination. Seropositivity among the vaccinated individuals is low probably because of single dose or insufficient time duration after vaccination in a limited number of HCWs/FLWs enrolled in the study. Seronegative cases indicate the possibility of absent, undetectable, or disappearing IgG antibodies and require further research.
